# Achieving elimination of soil-transmitted helminthiasis as a public health problem in Mali

**DOI:** 10.1136/bmjgh-2024-017092

**Published:** 2025-11-16

**Authors:** Mahamadou Traore, Fatoumata K Maiga, Modibo Keita, Boubacar Guindo, Moussa Sacko, Salif Seriba Doumbia, Mama Niele Doumbia, Benoit Dembele, Yaya Ibrahim Coulibaly, Cleo Stern, Steven Reid, Alexis Serna, Fiona M Fleming, Angela M Weaver, Yaobi Zhang

**Affiliations:** 1 Programme National de Lutte Contre les Schistosomiases et les Geohelminthes, Ministère de la Santé, Bamako, Mali; 2 Helen Keller International, Bamako, Mali; 3 National Institute of Public Health, Bamako, Mali; 4 Neglected Tropical Diseases Research Unit, Faculty of Medicine and OdontoStomatology, USTTB, Bamako, Mali; 5 Regional Office for Africa, Helen Keller International, Dakar, Senegal; 6 Helen Keller International, New York, NY, USA; 7 RTI International, Washington, DC, USA; 8 Unlimit Health, London, UK

**Keywords:** Public Health, Soil-transmitted helminth infections, Cross-sectional survey, Chemoprophylaxis, Health policy

## Abstract

**Introduction:**

Mali was endemic for soil-transmitted helminthiasis (STH), mainly hookworms in the southern regions. Following baseline mapping, mass drug administration (MDA) for STH was integrated with MDA for schistosomiasis for school-aged children (SAC) or lymphatic filariasis (LF) for populations aged five and older and vitamin A supplementation for preschool children. Epidemiological evaluations were conducted to assess progress towards eliminating STH as a public health problem.

**Methods:**

Cross-sectional studies were conducted in schools in 2004–2005 at baseline and in 2014–2019 for integrated evaluation with either schistosomiasis assessments or LF transmission assessment surveys (TAS). Children aged 7–14 years (6–7 years in TAS-STH surveys) were selected through systematic random sampling, and stool samples from selected children were examined using the Kato–Katz method for the eggs of any species of STH. The prevalence of infection and the prevalence of moderate-intensity and heavy-intensity (MHI) infections were calculated.

**Results:**

A total of 13 769 SAC were examined at baseline in 2004–2005, with an overall STH prevalence of 6.3% (95% CI 5.9% to 6.7%). Overall STH prevalence was the highest in Sikasso (22.9%), followed by Segou (9.4%). The prevalence of MHI infections ranged from 0% to 9.0% among the survey sites, with high prevalences (2.9–9.0%) in some communities in the Sikasso region. The predominant species of STH infection was hookworm, with negligible infection by *Ascaris lumbricoides* and *Trichuris trichiura*. Integrated schistosomiasis/STH impact assessments from 2014 to 2017 sampled 5776 children, with an overall prevalence of 0.1% (95% CI 0.1% to 0.3%) and 0% MHI infections. The integrated TAS-STH surveys in 2018–2019 in 29 districts further confirmed an overall low STH prevalence of 0.1% (95% CI 0.0% to 0.3%). These results indicate the progress towards the national goal of eliminating STH as a public health problem in Mali and highlight the need for continued surveillance in certain regions.

**Conclusion:**

Through over a decade of integrated treatment overcoming major security challenges, Mali may have successfully eliminated STH as a public health problem in all regions, one of the first countries in Africa to achieve this milestone.

**WHAT IS ALREADY KNOWN ON THIS TOPIC**

- Soil-transmitted helminth (STH) infections affect an estimated 1.5 billion people, including >265 million preschool-aged children and 633 million school-aged children, worldwide, mostly in the poorest and most deprived communities. In 2001, the World Health Assembly resolution (WHA54.19) urged Member States to sustain successful STH control efforts by regularly treating high-risk groups, especially school-aged children. WHO targets eliminating STH as a public health problem in almost all endemic countries by 2030 and recommends that endemic countries conduct epidemiological assessments to measure the success of their programmes.

**WHAT THIS STUDY ADDS**

- Despite a combination of drug donations from pharmaceutical companies and implementation funding from donors, global treatment coverage in children reached only 42.7% and 32.5% in Africa in 2020. Few countries have shown success in eliminating STH as a public health problem nationally. Mali scaled up the treatment and provided an example of such success in the most challenging environment.

**HOW THIS STUDY MIGHT AFFECT RESEARCH, PRACTICE OR POLICY**

- Mali’s experience in eliminating STH as a public health problem can be an example to other endemic countries in implementing and demonstrating a successful STH elimination programme.

## Introduction

 Soil-transmitted helminthiasis (STH) is caused by infection with a group of intestinal nematodes: roundworm (*Ascaris lumbricoides*), whipworm (*Trichuris trichiura*) and hookworm (*Necator americanus* and *Ancylostoma duodenale*).^1 2^ Globally, an estimated 1.5 billion people are infected with one or more of these parasites, typically affecting communities with poor access to clean water, sanitation and hygiene in tropical and subtropical areas.^2^ The World Health Assembly resolution (WHA54.19) urged Member States to sustain successful STH control efforts by providing regular treatment to high-risk groups, especially school-aged children (SAC), with a minimum treatment coverage of 75% by 2010.^3^ Despite the efforts by endemic countries, the global minimum target was not reached by 2010. In 2012, the WHO 2020 Roadmap for Neglected Tropical Diseases (NTDs) set the goal to achieve and sustain this minimum coverage of 75% in preschool children and SAC by 2020 in all countries where STH was considered a public health problem (defined by a threshold of 1% of moderate-intensity and heavy-intensity (MHI) infections in SAC).^4 5^ The 2020 Roadmap prompted increased drug donations from pharmaceutical companies and implementation funding from donor countries through international organisations, leading to improved treatment coverage—42.7% globally and 32.5% in Africa in 2020.^6 7^ Following this scale-up, the prevalence of STH among SAC in sub-Saharan Africa was significantly reduced from the pre-2000 levels.^8 9^ Building on this success, the WHO Roadmap 2021–2030 set a target of eliminating STH as a public health problem, redefined as <2% proportion of MHI infections, in all endemic countries by 2030.^10 11^ As of 2022, an estimated 265.3 million preschool children, 632.6 million SAC, 108 million adolescent girls and 138.8 million pregnant and lactating women in 87 countries worldwide require deworming treatment for STH.^12^

**Figure 1:**
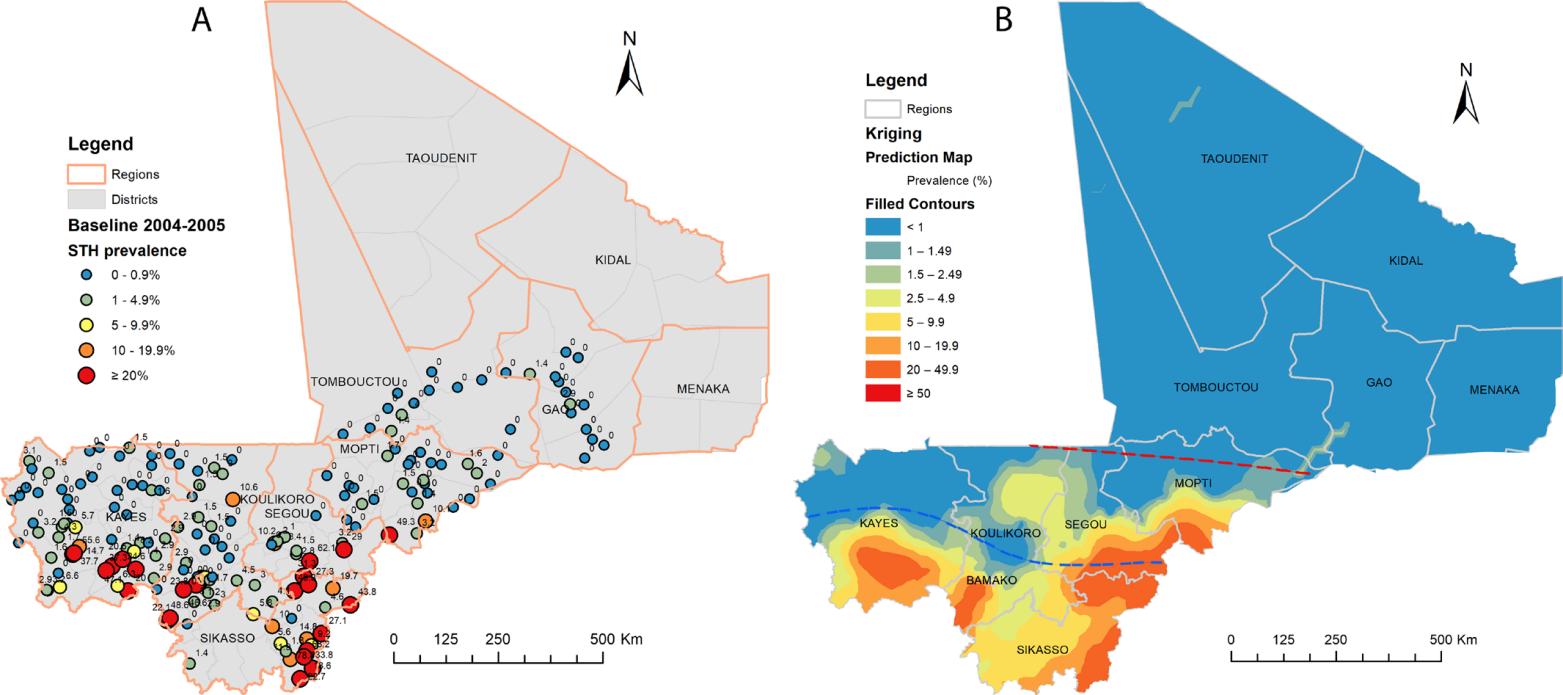
Distribution of soil-transmitted helminthiasis (STH) point prevalence (A) and spatially smoothed contour maps of predicted STH prevalence (B) at baseline in 2004–2005 in Mali. Three sites, one in Mopti (11.9%) and two in Segou (both 0%), were not plotted due to missing global positioning system coordinates. Red and blue dotted lines on (B) represent approximate divisions of three climate zones (ref #13).

Mali is a landlocked country in West Africa with three climate zones (figure 1).^13^ The northern zone lies deep in the Sahara Desert, with an extremely hot desert climate and low rainfall (<50 mm) throughout the year. The central zone has a hot semi-arid climate with a long dry season and a short, irregular rainy season. The southern zone has a tropical wet and dry climate with annual rainfall of 700–1400 mm.^14 15^ Surveys before 2000 found that STH was primarily due to hookworm infection, with *A. lumbricoides* and *T. trichiura* infections being rare. Hookworm infection was mainly prevalent in the regions of the southern climate zone and was not endemic in the regions within the northern desert climate zone.^816^,^18^ The distribution of hookworm infection in Mali was closely related to the annual rainfall in each region and overlapped with the distribution of schistosomiasis.^16^ The focal prevalence of hookworm infection in Mali’s southernmost region, Sikasso, has been reported to be as high as 70%.^17 18^

STH control in Mali consisted of three main drug delivery approaches.^19 20^ First, following baseline mapping surveys, integrated schistosomiasis/STH treatment was conducted (2005–2007), with albendazole (ALB) and praziquantel through school-based and community-based drug delivery targeting children aged 5–14 years. The frequency of STH treatment followed the frequency of schistosomiasis treatment in different districts depending on schistosomiasis prevalence thresholds. Second, Mali started annual mass drug administration (MDA) for lymphatic filariasis (LF) elimination in 2005, initially in four co-endemic onchocerciasis districts in Sikasso using community-directed treatment with ivermectin plus ALB. All LF MDA-eligible populations (≥5 years) benefited from annual MDA for STH treatment. In 2008, the national integrated NTD programme started, and STH treatment became part of the coordinated drug administration for all five NTDs targeted by preventive chemotherapy, principally through LF MDA.^20^ The LF MDA was progressively scaled up to national coverage of all health districts in 2009, then gradually scaled down as elimination of LF as a public health problem was achieved in each district and eventually stopped across the country by 2020. In total, STH-endemic districts received 7–11 rounds of treatment each through LF MDA. Third, the National Intensified Nutrition Weeks (SIAN, French acronym) provide ALB along with vitamin A capsules twice a year to children aged 12–59 months while also treating women immediately after childbirth. Deworming through SIAN started in 2006 and is ongoing.

Integrated impact assessments were conducted in 2014–2017 in all STH-endemic regions through schistosomiasis/STH sentinel site surveys and in 2018–2019 through the LF transmission assessment survey (TAS). The results showed that STH may no longer be a public health problem in Mali. This article presents the results of the surveys and discusses the further needs for STH treatment and surveillance plans.

## Methods

### Baseline survey

National integrated schistosomiasis/STH baseline mapping was conducted in 2004–2005.^21 22^ The survey strategy was previously described.^21^ Schools were selected at baseline in regions previously identified as endemic for schistosomiasis/STH, primarily in the southern and central climate zones.^16^ A one-decimal-degree squared grid was overlaid over the country in ArcView, and communities were randomly selected from each grid cell. In each school, 30 boys and 30 girls aged 7–14 years were selected by systematic random sampling. For each selected child, a single faecal sample was collected and examined using the standard Kato–Katz method. Two Kato–Katz slides were prepared from each faecal sample and examined microscopically within 1 hour of preparation by trained laboratory technicians. Experienced microscopists re-read 10% of the slides from each technician for quality control. The number of STH eggs was counted and recorded from two slides. The mean egg count from two slides was calculated for each child and expressed as eggs per gram of faeces (epg). Geographic locations of schools were recorded using a handheld global positioning system device.

### Assessment through sentinel sites

Between 2014 and 2017, a school-based integrated impact assessment for schistosomiasis/STH was conducted in all STH-endemic regions. Survey sites were purposefully selected in 40 health districts across five regions (Sikasso, Koulikoro, Kayes, Segou and Mopti) and the capital district of Bamako, with 1–4 sites per district. At each survey site/school, 60 children (30 boys and 30 girls) aged 7–14 years old were systematically randomly selected. A single faecal sample was collected from each selected child and examined as described above. One Kato–Katz slide was used per sample. The egg count from one slide was calculated and expressed as epg for each child.

### Assessment through LF TAS

WHO recommends the assessment of STH infections during LF TAS.^23^ In 2018–2019, an LF TAS-STH survey was conducted in 29 health districts in Kayes, Koulikoro, Sikasso and Bamako using the WHO-recommended survey protocol.^23^ Twenty-nine (29) health districts were grouped into 11 evaluation units (EUs) according to their programmatic and epidemiological situation. In each EU, an exhaustive list of villages (clusters) was compiled, and approximately 30 clusters were randomly selected using the TAS-STH Survey Sample Builder (SSB).^24^ For the concurrent STH survey, a subset of 332 children aged 6–7 years, a sample size predefined in the WHO recommendations,^23^ was sampled in the same villages (clusters) as the LF TAS using a random list generated by the SSB. A single stool sample was collected from each selected child, with one Kato–Katz slide used per sample. Egg counts for STH infections were recorded and expressed as epg.

### Data analysis

Descriptive analysis of the data sets was performed using SPSS Statistics (IBM, V.23). The intensity of infection of each child was categorised as light-intensity, moderate-intensity or heavy-intensity infection for each species using the epg of each child according to the WHO categorisation.^25^ The prevalence with 95% CI and the prevalence of MHI infections (with range) were calculated by region. For the TAS-STH surveys, the number of children with STH infection was assessed against the predetermined critical cut-off values for each EU, and the prevalence threshold for each EU was then applied according to the WHO guidelines.^23^ Survey sites and prevalence maps were created using ArcGIS V.10.8.2 (ESRI, Redlands, USA). Geostatistical modelling of point prevalence was performed using ordinary kriging with a stable semivariogram model in ArcGIS Geostatistical Analyst to predict the prevalence distribution of STH at baseline and in 2014–2017. Spatial aggregation of STH infections was analysed using the spatial autocorrelation (Moran’s *I*) tool, and locations of such a local aggregation of STH infections were identified using the hotspot analysis (Getis-Ord Gi∗) tool in ArcGIS.

### Patient and public involvement

The survey designs followed WHO standard recommendations. Communities (village chiefs, school teachers, parents of children or children recruited) or the public were not involved in the design of the surveys but were involved in conducting the surveys and reporting and disseminating the survey results in their respective communities.

## Results

### Baseline results in 2004–2005

**Figure 2:**
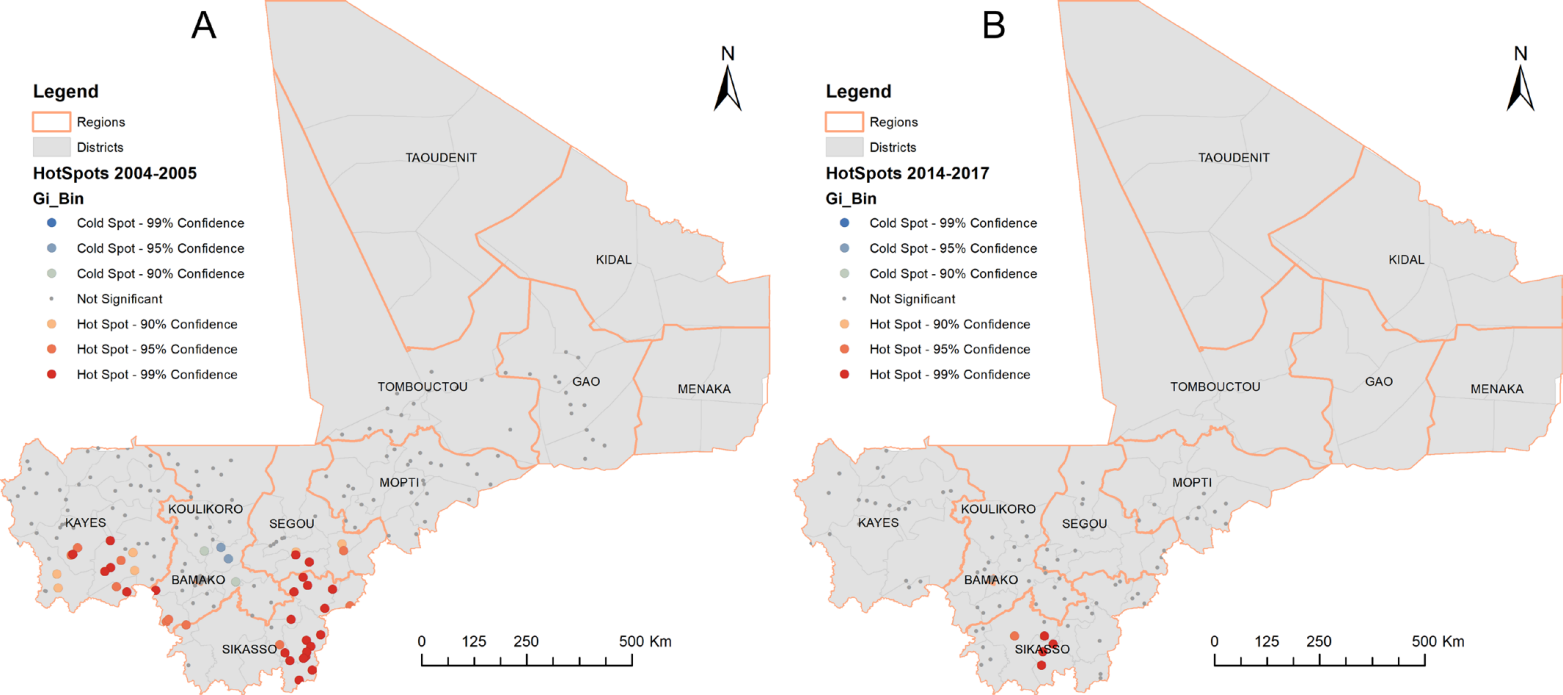
Local spatial aggregation of soil-transmitted helminthiasis infections in Mali in 2004–2005 (A) and 2014–2017 (B).

At baseline (2004–2005), 199 sites were surveyed across seven regions and Bamako capital district, with a total of 13 769 children (7–14 years) examined. Table 1 summarises the prevalence of STH at baseline. The overall prevalence of STH in SAC was 6.3% (95% CI 5.9% to 6.7%) in the areas surveyed. The Sikasso region had the highest prevalence of STH (22.9%), followed by the Segou region (9.4%). High community prevalence of ≥20%, ranging from 20.0% to 78.8%, was found in the regions of Sikasso, Segou, Kayes, Koulikoro and Mopti (figure 1A). Spatial analysis showed that the predicted prevalence distribution was in line with the southern and central climate zones (figure 1B). The spatial distribution of STH infections showed a significant spatial clustering (Moran’s *I*: 0.2167; expected Moran’s *I*: −0.0051; variance: 0.0045; *Z*-score: 3.3017, p=0.0010), with local aggregation of STH prevalence distributed in the Sikasso, Segou, Kayes and Koulikoro regions (figure 2A). The overall prevalence of MHI infections was low (0.1%) and ranged from 0% to 9.0% across the survey sites. High prevalence of MHI infections (>2%) was found in certain communities in the Sikasso region, ranging from 2.9% to 9.0%.

**Table 1:** Prevalence of soil-transmitted helminthiasis infections by regions in 2004–2005 in Mali

Region	Number of children tested	Number of children positive for hookworm	Prevalence (%) of hookworm infection (95% CI)	Number of children positive for *A. lumbricoides*	Prevalence (%) of *A. lumbricoides* infection (5% CI)	Number of children positive for *T. trichiura*	Prevalence (%) of *T. trichiura* infection (95% CI)	Number of children with any infection	Prevalence (%) of any species infection	Median prevalence (%) of any species infection (range)	Number of children with MHI infections	Prevalence (%) of MHI infections (range)
Bamako	1322	14	1.1 (0.6 to 1.8)	0	0 (0 to 0.3)	4	0.3 (0.1 to 0.8)	18	1.4 (0.9–2.1)	1.1 (0–4.6)	1	0.1 (0–1.4)
Gao	968	0	0 (0 to 0.4)	1	0.1 (0 to 0.6)	2	0.2 (0.1 to 0.8)	3	0.3 (0.1–0.9)	0 (0–2.9)	0	0.0
Kayes	3471	213	6.1 (5.4 to 7.0)	4	0.1 (0 to 0.3)	4	0.1 (0 to 0.3)	214	6.2 (5.4–7.0)	1.4 (0–55.6)	1	0.0 (0–1.5)
Koulikoro	2506	131	5.2 (4.4 to 6.2)	2	0.1 (0 to 0.3)	7	0.3 (0.1 to 0.6)	136	5.4 (4.6–6.4)	1.5 (0–48.6)	0	0.0
Mopti	2048	55	2.7 (2.1 to 3.5)	2	0.1 (0 to 0.4)	4	0.2 (0.1 to 0.5)	58	2.8 (2.2–3.6)	0 (0–49.3)	0	0.0
Segou	1246	116	9.3 (7.8 to 11.1)	0	0 (0 to 0.3)	2	0.2 (0 to 0.6)	117	9.4 (7.9–11.1)	2.0 (0–62.1)	3	0.2 (0–1.9)
Sikasso	1378	313	22.7 (20.6 to 25.0)	8	0.6 (0.3 to 1.1)	9	0.7 (0.3 to 1.2)	315	22.9 (20.7–25.2)	18.6 (0–78.8)	13	0.9 (0–9.0)
Tombouctou	830	0	0 (0 to 0.5)	0	0 (0 to 0.5)	2	0.2 (0.1 to 0.9)	2	0.2 (0.1–0.9)	0 (0–1.4)	0	0.0
Overall	13 769	842	6.1 (5.7 to 6.5)	17	0.1 (0.1 to 0.2)	34	0.2 (0.2 to 0.3)	863	6.3 (5.9–6.7)	1.2 (0–78.8)	18	0.1 (0–9.0)

MHI, moderate and heavy intensity.

The predominant species of STH was hookworm, with an average prevalence of infection of 6.1% (95% CI 5.7% to 6.5%). Infections with *A. lumbricoides* and *T. trichiura* were rare with very low prevalence in these regions (table 1).

### Impact assessment results between 2014 and 2019

**Figure 3:**
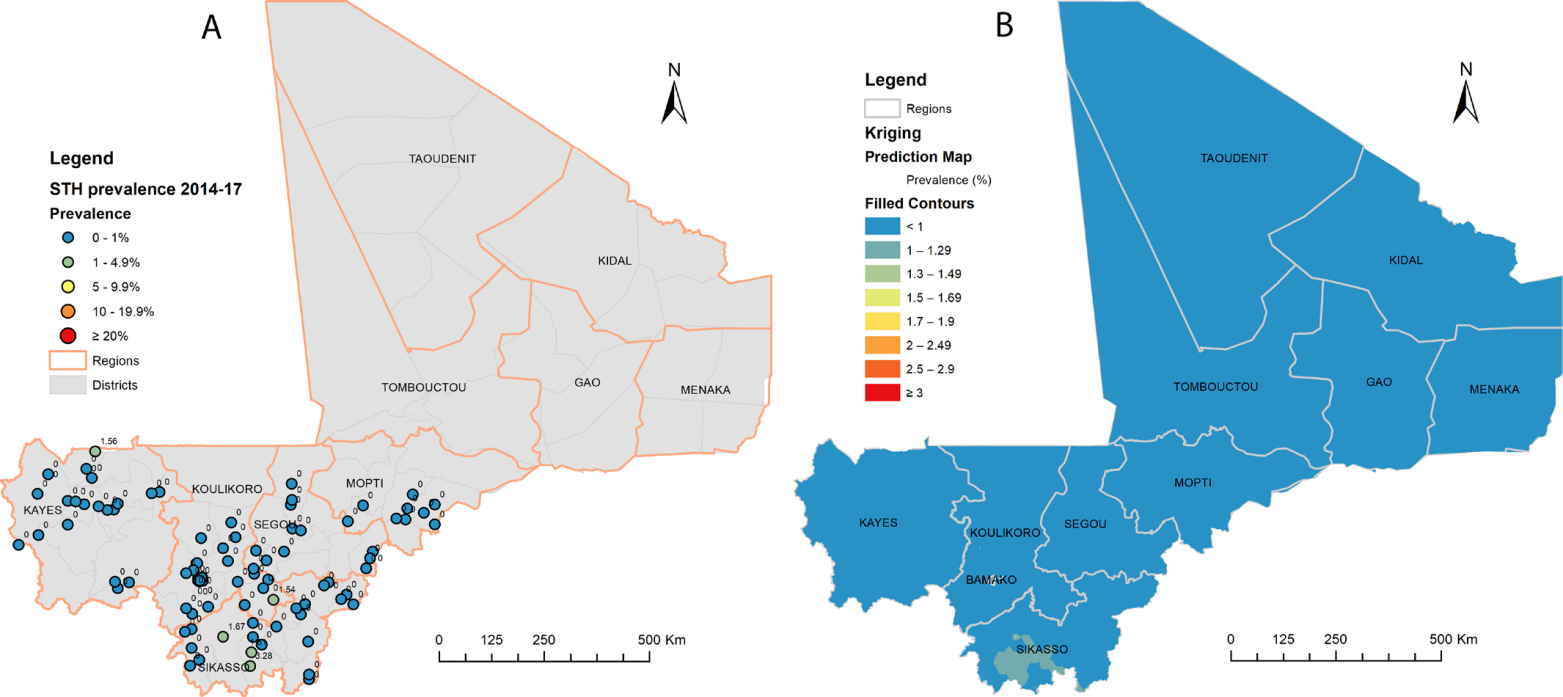
Distribution of soil-transmitted helminthiasis (STH) point prevalence (A) and spatially smoothed contour maps of predicted STH prevalence (B) at impact assessments in 2014–2017 in Mali.

Between 2014 and 2017, impact assessments examined 5776 children at 95 sites in 40 health districts in five regions and Bamako capital district. STH infection was detected in seven sites. Four cases of hookworm infection were found in the Sikasso region, and one case of *T. trichiura* infection each was found in the regions of Kayes and Koulikoro and Bamako capital district (table 2). No *A. lumbricoides* infection was detected. The overall prevalence of STH infection in SAC was 0.1% (95% CI 0.1% to 0.3%). None of the infected children had MHI infections: 0% (95% CI 0% to 0.1%). Among 95 sites across the surveyed areas, the prevalence of STH infection ranged from 0% to 3.3% (median prevalence: 0%). Only part of Sikasso had residual STH infection with very low prevalence (figure 3). Spatial clustering of STH infections seen at baseline was no longer shown (Moran’s *I*: 0.0601: expected Moran’s *I*: −0.0106; variance: 0.3754; Z-score: 0.1155, p=0.9080). The hotspot analysis showed potential local aggregation of residual STH infections in SAC in the Sikasso region, specifically in Kolondieba and Bougouni districts (figure 2B).

**Table 2:** Prevalence of soil-transmitted helminthiasis infections in regions surveyed in 2014–2017 in Mali

Regions	Number of children tested	Number of children positive for hookworm	Prevalence (%) of hookworm infection	Number of children with *T. trichiura* infection	Prevalence (%) of *T. trichiura* infection	Prevalence (%) of any species infection
Bamako	719	0	0 (0–0.5)	1	0.1 (0–0.8)	0.1 (0–0.8)
Kayes	1300	0	0 (0–0.3)	1	0.1 (0.0–0.4)	0.1 (0.0–0.4)
Koulikoro	1037	0	0 (0–0.4)	1	0.1 (0.0–0.5)	0.1 (0.0–0.5)
Mopti	608	0	0 (0–0.6)	0	0 (0–0.6)	0 (0–0.6)
Segou	729	0	0 (0–0.5)	0	0 (0–0.5)	0 (0–0.5)
Sikasso	1383	4	0.3 (0.1–0.74)	0	0 (0–0.3)	0.3 (0.1–0.7)
Total	5776	4	0.1 (0.0–0.2)	3	0.1 (0.0–0.2)	0.1 (0.1–0.3)

**Figure 4:**
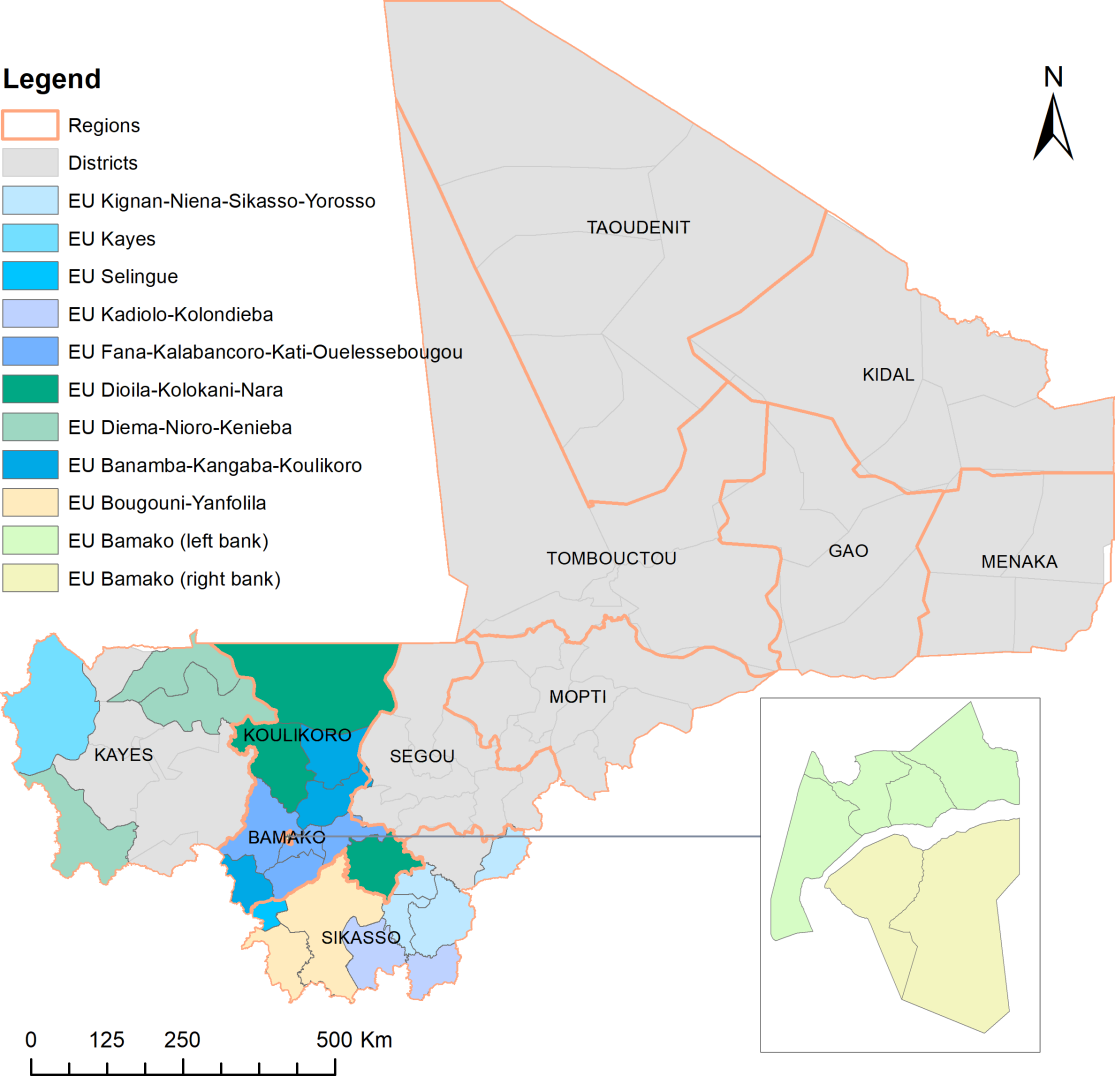
Distribution of 11 evaluation units across 29 health districts in Kayes, Koulikoro, Sikasso and Bamako in 2018–2019. EU, evaluation unit.

In 2018–2019, the STH assessment was integrated into the LF TAS in 11 EUs covering 29 health districts across Kayes, Koulikoro, Sikasso and Bamako. The distribution of EUs is shown in figure 4. There were two cases of *A. lumbricoides* infection and two cases of hookworm infection among 3810 children tested in all 11 EUs, with an overall prevalence of 0.1% (95% CI 0.0% to 0.3%) (table 3). According to the critical cut-off values defined in the WHO guidelines, eight EUs were defined as STH prevalence<2%, and three EUs were defined as STH prevalence 2 to <10%. There were no MHI infections observed, and the overall prevalence of MHI infections was 0% (95% CI 0% to 0.1%). Using the number of MHI infections in each EU against the critical cut-off values, the prevalence of MHI infections was defined as <2% in all 11 EUs.

**Table 3:** Prevalence thresholds of soil-transmitted helminthiasis infections in evaluation units in 2018–2019 in Mali

Evaluation units	Number of children tested	Number of children tested positive	Prevalence (%) threshold	Number of children with MHI infections	MHI prevalence (%) threshold
Selingue	410	0	<2	0	<2
Kignan–Niena–Sikasso–Yorosso	350	0	<2	0	<2
Kadiolo–Kolondieba	324	0	<2	0	<2
Kayes	345	0	<2	0	<2
Fana–Kalabancoro–Kati–Ouelessebougou	347	1	2 to <10	0	<2
Dioila–Kolokani–Nara	348	1	2 to <10	0	<2
Diema–Nioro–Kenieba	342	0	<2	0	<2
Banamba–Kangaba–Koulikoro	341	2	2 to <10	0	<2
Bougouni–Yanfolila	343	0	<2	0	<2
Bamako (left bank)	273	0	<2	0	<2
Bamako (right bank)	387	0	<2	0	<2
Total	3810	4	0.1 (0.0–0.3)	0	0 (0–0.1)

MHI, moderate and heavy intensity.

## Discussion

In Mali, after over a decade of treatment interventions, STH infections were rarely seen in SAC, with no MHI infections detected (0%) in each region and survey site during the 2014–2019 impact assessment surveys. WHO defined the target for eliminating STH as a public health problem when the prevalence of MHI infections was <1% in SAC previously^5^ and <2% in children currently.^10 11^ Judged by either criterion, the results of the impact assessments suggest Mali has successfully achieved the goal of eliminating STH as a public health problem in each endemic region, becoming one of the first countries in Africa to achieve this milestone, pending validation by WHO (WHO guidance on validation is yet to be developed).

The baseline mapping results showed spatial clustering and local aggregation of STH infections in the southern climate zone of the country (figures1  2A). STH transmission requires a warm and moist environment,^26^ and extreme climate and temperature are detrimental to the survival of the free-living stage of the parasites.^27^ The extreme climate (dry and hot) in the northern part of Mali is not favourable to STH transmission. The results were consistent with the previous survey findings in Mali.^16^,^18^ The overall prevalence of STH in the regions surveyed at baseline was relatively low at 6.3%, but in some communities, particularly in the Sikasso region, hookworm prevalence was close to 80%, and the prevalence of MHI infections was up to 9.0%. Hookworm was the predominant species of infection, and infection with *A. lumbricoides* or *T. trichiura* was negligible, similar to findings from other countries in West Africa.^8 9 28 29^

A recent analysis from the geostatistical modelling showed that across sub-Saharan Africa, the overall prevalence of STH in children aged 5–14 years was reduced from 44% in 2000 to 13% in 2018 due to the sustained use of preventive chemotherapy, improved sanitation and economic development.^9^ However, there were still a significant number (25%) of implementation units with the prevalence of MHI infections>2%.^9^ Mali’s impact assessment results presented here were in line with the overall trend of a decrease in STH prevalence in Africa. A low prevalence of STH infection after years of MDA has also been reported in Burkina Faso and Senegal.^28 30 31^ This contrasts with results from some other countries, such as Nigeria,^32^ Tanzania^33 34^ and Uganda,^35 36^ where STH prevalence remained high. Environmental factors such as the Sahelian conditions and limited geographical distribution, with a relatively low prevalence of STH at baseline, may have facilitated such an achievement in Mali. In the absence of guidance from WHO on the next phase of the programme to eliminate STH transmission, the National Expert Committee on Schistosomiasis and STH in 2021, in consultation with WHO, recommended that STH MDA in SAC was no longer necessary. It was noted that three EUs (10 health districts) were classified as having STH prevalence of 2 to <10%, which may require treatment of SAC every other year as per WHO guidelines. However, considering the very low prevalence and no MHI infections from the sentinel site assessments in the same regions, the national programme concluded that it was not necessary to restart the STH MDA in SAC after LF MDA stopped. Therefore, STH MDA in SAC was suspended, and STH was placed under surveillance.^6 11^

Although STH MDA is suspended in SAC, there were still residual STH infections in SAC with local aggregation in Sikasso as shown in figures2B  3. Therefore, some case treatment should be provided at local health facilities or through school health programmes, and drugs should be made available for this purpose. Currently, the deworming of children under five and women immediately after childbirth is still ongoing during SIAN. It is important to continue these efforts to avoid a resurgence of infection. In addition, the prevalence of anaemia in children under 5 years of age remains high in Mali,^37^ and deworming this group of children could help alleviate the widespread problem of anaemia. However, programme survey data on STH prevalence and geographical distribution must be considered when deciding on deworming. Deworming through SIAN should focus on areas in southwest Mali, particularly in the Sikasso region, while deworming in other areas, particularly in northern Mali during SIAN, is unlikely to be necessary.

The prevalence of hookworm infection increases with age, with more infections in adults than in SAC.^2938^,^40^ The status of hookworm infection in Malian adults has not yet been assessed through any impact surveys. As mass treatment of adults under the LF MDA has been stopped in Mali, the national programme should take necessary measures to provide treatment to older at-risk groups, particularly women of childbearing ages.

The national programme must establish a surveillance system and continue to monitor the situation to prevent recrudescence, given the situation in SAC and adults discussed above. WHO has recently published a monitoring and evaluation framework for schistosomiasis and STH.^41^ The recommended surveillance strategies should be adopted in Mali. Given the fact that the LF TAS is being phased out and will soon no longer be available in Mali to assess STH in children, a decentralised and integrated monitoring system through sentinel sites should be established, focusing on areas with the highest risk of rebound as recommended by WHO.^41^ Schistosomiasis/STH assessment surveys are currently being implemented and expanded throughout Mali. The current survey uses a district cluster survey protocol. Initial results in SAC from such surveys in 2023 in eight districts in Kayes, Koulikoro, Segou and Bamako suggest that elimination of STH as a public health problem has been maintained.^42^ However, none of these surveys provide information on STH infection in the adult population. It is therefore proposed to include adults in STH surveys at a few sites in the former high hookworm prevalence villages in the Sikasso region to assess the hookworm situation in adults. National STH surveillance should also include passive disease reporting from local health facilities through the district health information system for any signal of STH cases. Appropriate action should be taken to investigate further if there is any increase in STH case reports through the national health system, paying attention to potential high-risk areas of residual STH infection in the Sikasso region. In addition, access to safe water, sanitation and hygiene (WASH) facilities is critical to the control and elimination of STH^43^ and is particularly important to sustain the impact of control and maintain elimination gains in the long term after STH treatment is scaled down or stopped.^44^ The national programme has been coordinating with the WASH sector, and any measures put in place for surveillance and coordination with WASH intervention would help maintain the gains made in STH elimination in Mali.

Since 2011, Mali has experienced multiple challenges, namely political and social instability and insecurity with terrorist attacks, which seriously affected the implementation of NTD programme activities. The national NTD programme, in collaboration with communities, donors and supporting partners, implemented adaptive strategies in insecure areas to maintain MDA coverage and conduct field assessment surveys when needed. In areas with security challenges, training of local trainers and local supervisors for MDA and training of local health centre staff for surveys were conducted in safe locations outside insecure zones. Following training in safe district towns, community drug distributors implemented MDA in their respective communities under local supervision. Local health centre staff conducted surveys in their local context with remote supervision from the central team. Such adaptive approaches have supported Mali in meeting many of its NTD elimination targets.

There were certain limitations of the studies. First, the survey methodologies for impact assessments were different from those at baseline, and the impact results could not be statistically compared with the baseline data. However, all impact surveys were conducted according to the WHO recommendations, and therefore, the results should represent the epidemiological situation in the regions. Second, one Kato–Katz slide on a single faecal sample was used in the impact surveys. The sensitivity of such tests is low in areas with low prevalence and low intensity of infection.^45 46^ The STH prevalence presented may have been underestimated, but such tests should be able to detect MHI infections, if any. The conclusion of the article should not be affected as the prevalence of MHI infections is the critical indicator for eliminating STH as a public health problem.

In conclusion, by integrating STH treatment with schistosomiasis MDA, LF MDA and vitamin A supplementation and overcoming political, social and security challenges, Mali may have successfully eliminated STH as a public health problem, one of the first countries in Africa to achieve this milestone (pending on the WHO validation). Surveillance has been put in place to ensure that the gains are maintained.

## Data Availability

Data are available upon reasonable request.
